# Nitrofurantoin-Induced Lung Injury: A Reminder of an Overlooked Threat

**DOI:** 10.7759/cureus.44730

**Published:** 2023-09-05

**Authors:** Aasir M Suliman, Mohammed A Alamin, Irfan Ul Haq

**Affiliations:** 1 Pulmonology, Hamad Medical Corporation, Doha, QAT; 2 Internal Medicine, Hamad Medical Corporation, Doha, QAT

**Keywords:** drug-induced lung injury, ild, interstitial lung disease, lung injury, nitrofurantoin

## Abstract

Nitrofurantoin is a commonly prescribed antibiotic for urinary tract infection (UTI) treatment and prophylaxis. Although relatively rare, nitrofurantoin can cause a spectrum of lung injuries, from acute hypersensitivity reactions that might be fatal to chronic reactions involving fibrosis. Therefore, treating physicians’ awareness and regular monitoring is essential for early recognition, drug withdrawal, avoiding unnecessary treatment, and preventing irreversible fibrosis. Here, we report the case of a 77-year-old woman who had been hospitalized with interstitial lung disease due to chronic nitrofurantoin therapy. Despite the severity of symptoms and the extent of radiological lung involvement, she returned to her clinical and radiological baseline shortly following the drug cessation.

## Introduction

Nitrofurantoin is a well-known antibacterial agent used in treating urinary tract infections (UTIs), particularly acute uncomplicated cystitis, and is also used in prophylaxis for patients with recurrent UTIs [1,2]. Pulmonary toxicity to nitrofurantoin has been increasingly reported in the literature, and it can be either an acute or chronic reaction [3]. These reactions are generally reversible with a good prognosis; however, potentially severe and even fatal pulmonary adverse reactions may occur, necessitating prompt recognition and early drug elimination [3,4]. Here, we present the clinical course of an elderly female who developed interstitial lung disease necessitating hospitalization for hypoxic respiratory failure six months after starting nitrofurantoin. 

## Case presentation

A 77-year-old woman presented to the emergency department with a four-week history of progressive shortness of breath and dry cough. She had no fever, orthopnea, paroxysmal nocturnal dyspnea, chest pain, or recent upper respiratory tract infection. She denied any rheumatological/vasculitis symptoms, and the other systems review was unremarkable. Her past medical history included hypertension, vitiligo, and left breast cancer treated with bilateral mastectomy and left axillary clearance (without radiotherapy). She had been taking nitrofurantoin for recurrent UTIs for the past six months. She was a housewife with no prior occupational exposure and a lifetime non-smoker. She had no animals, farm, or any concerning indoor exposure. 

**Figure 1:**
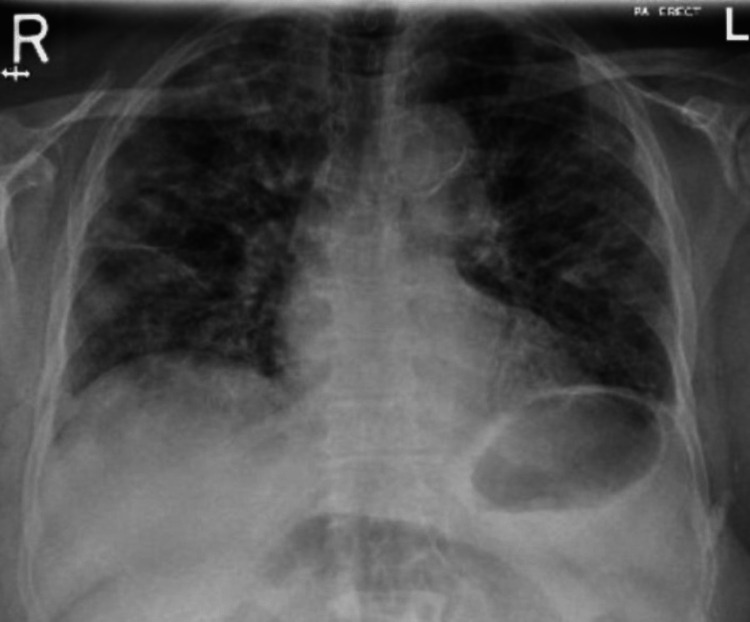
Chest X-ray showing bilateral reticular infiltrates with patchy ground glass opacities

On examination, she was afebrile, tachypneic with a respiratory rate of 28 breaths per minute, and required oxygen of 4-5 liters per minute via nasal cannula to maintain oxygen saturation above 94%. Chest auscultation was remarkable for fine bibasilar crackles. The initial blood labs showed mild normocytic normochromic anemia (hemoglobin (Hb) 11.0 mg/dl), normal renal and liver chemistry, N-terminal pro-b-type natriuretic peptide (NT-pro BNP) level of 421 pg/ml, and high inflammatory markers. An echocardiogram showed normal left ventricle ejection fraction with grade 3 diastolic dysfunction, and the chest x-ray (CXR) revealed bilateral reticular infiltrates with patchy ground glass opacities (Figure 1). She was admitted for hypoxic respiratory failure with a working diagnosis of community-acquired pneumonia and possible decompensated heart failure; accordingly, she was commenced on intravenous broad-spectrum antibiotics and diuretics.

**Figure 2:**
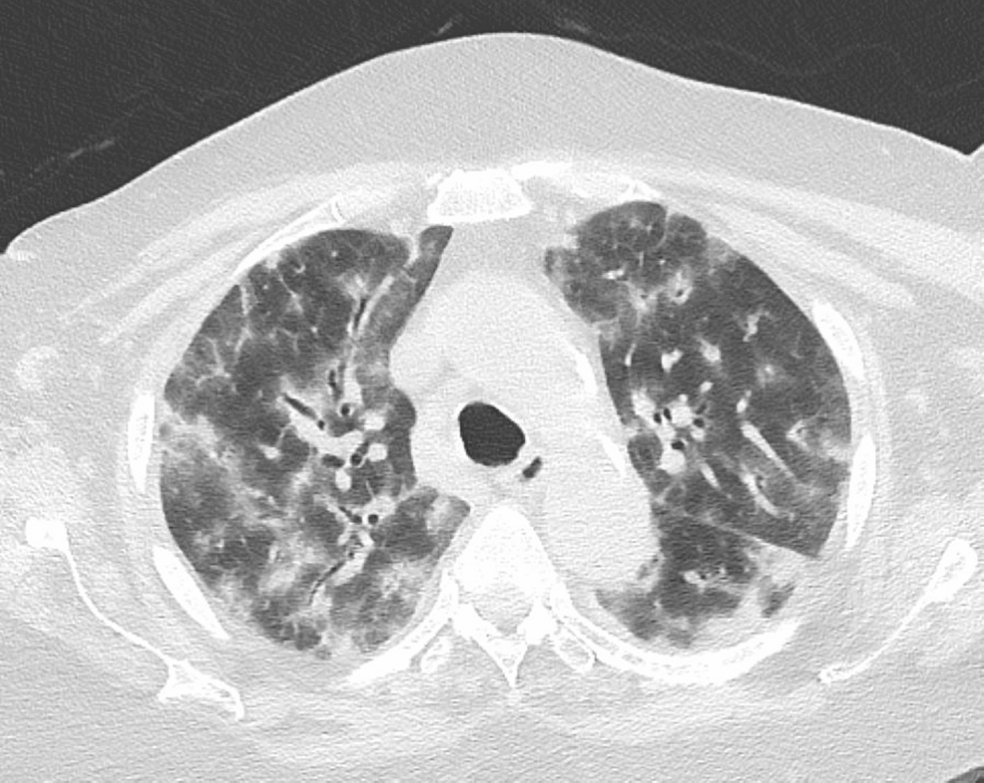
CT thorax showing bilateral patchy ground glass opacities associated with interlobular septal thickening, peri bronchial consolidations and traction bronchiectasis (upper lobes)

**Figure 3:**
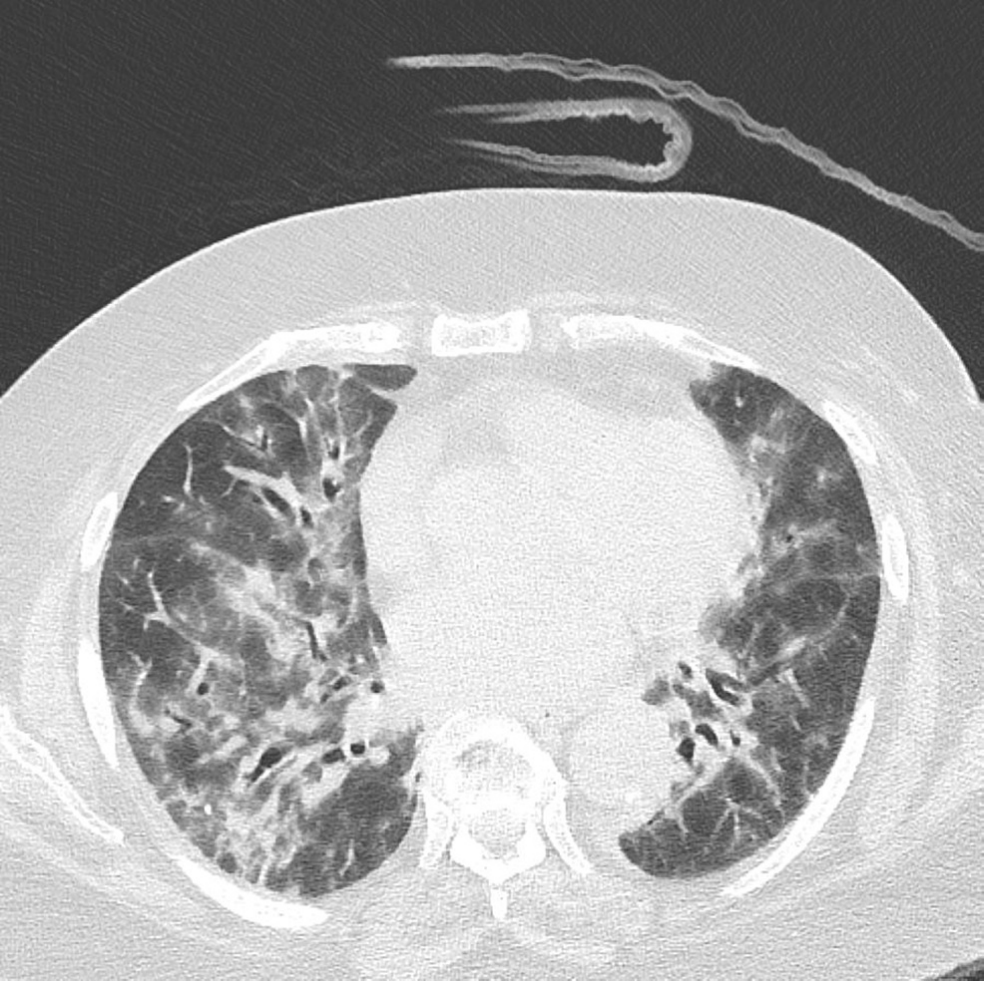
CT thorax showing bilateral patchy ground glass opacities associated with interlobular septal thickening, peri bronchial consolidations, and traction bronchiectasis (middle and lower lobes)

Computed tomography (CT) scan of the thorax showed diffuse patchy bilateral ground-glass opacities associated with bilateral interlobular septal thickening, peribronchial consolidations, and traction bronchiectasis; features suggestive of interstitial lung disease (Figures 2-3). Pulmonary function test revealed a restrictive pattern with decreased lung volumes and diffusion. Subsequently, a flexible bronchoscopy with bronchoalveolar lavage (BAL) was performed, and an autoimmune screen was sent. BAL microbiology work-up was negative; the cell count was as follows: neutrophils 67%, lymphocytes 16%, macrophages 17%, and eosinophils 0%. The autoimmune work-up was negative.

**Figure 4:**
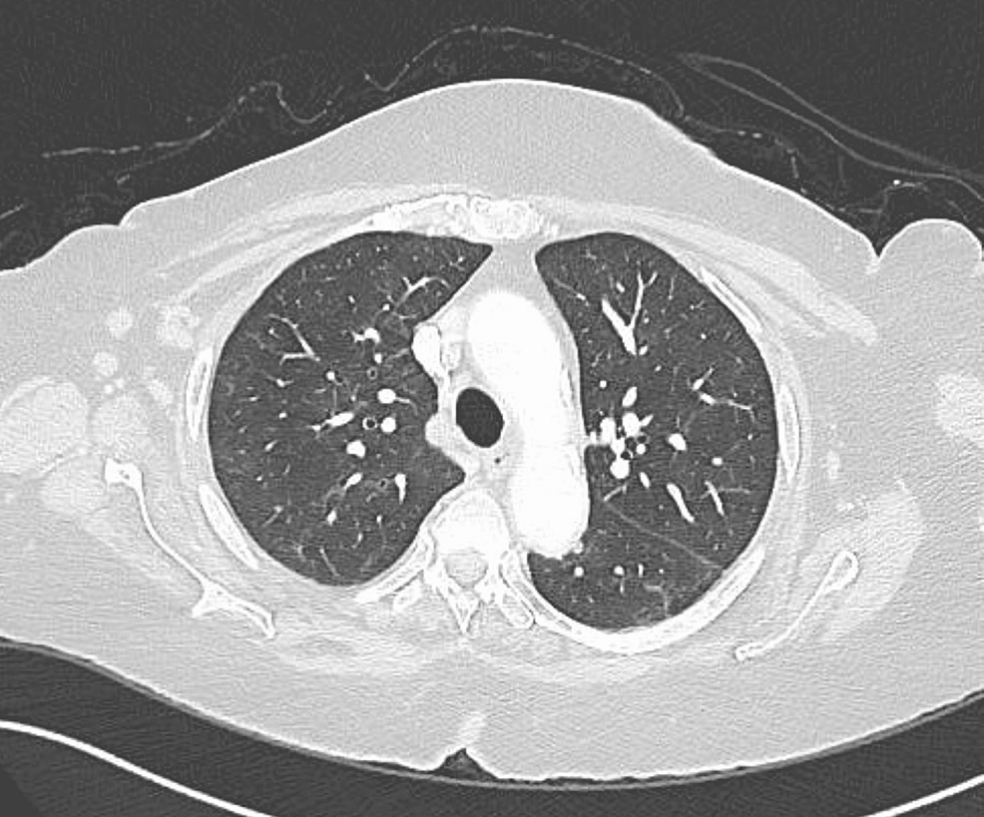
A follow-up CT thorax after four weeks showing almost complete resolution of the reticular and ground glass infiltrates with a residual mild traction bronchiectasis (upper lobes)

**Figure 5:**
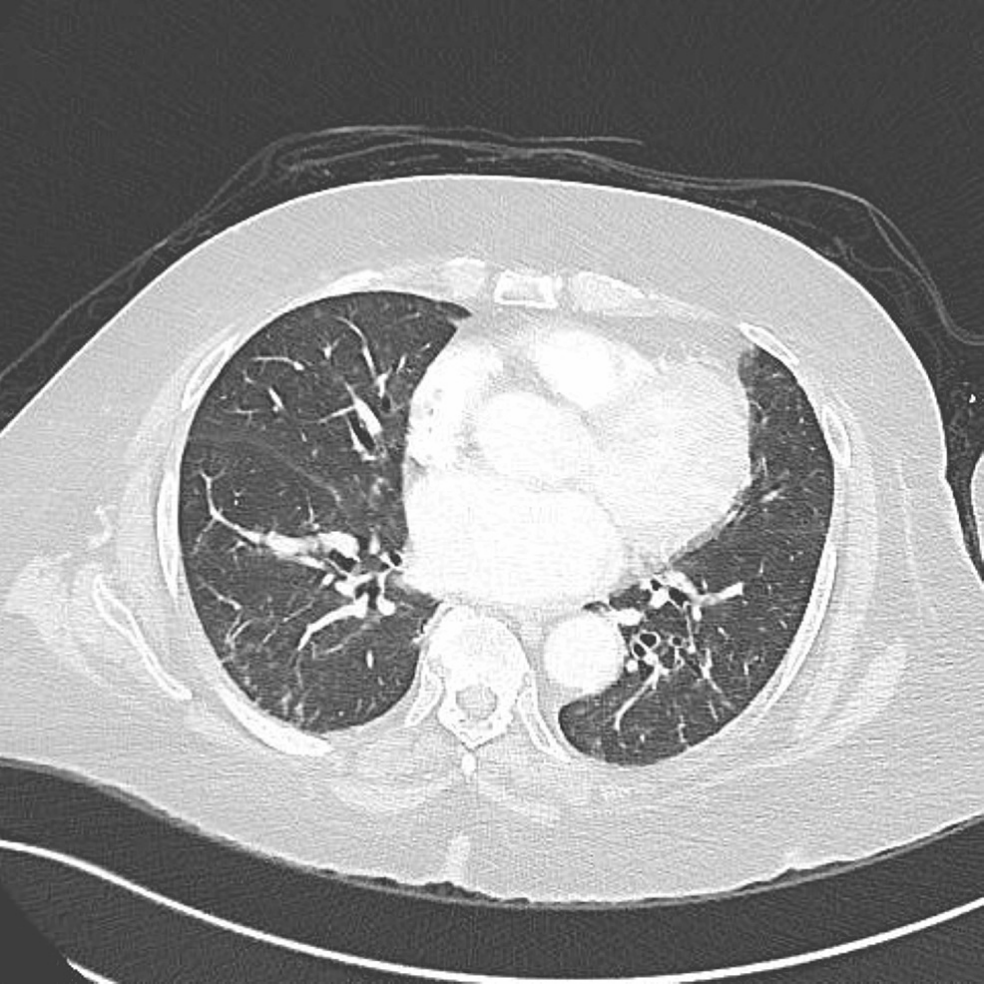
A follow-up CT thorax after four weeks showing almost complete resolution of the reticular and ground glass infiltrates with a residual mild traction bronchiectasis (middle and lower lobes)

Based on a compatible history of exposure to nitrofurantoin, radiological findings of interstitial lung disease, and restrictive lung physiology, a diagnosis of nitrofurantoin-induced interstitial lung disease was suspected, and nitrofurantoin was withdrawn. Therefore, a lung biopsy was not done. Within two weeks of admission, the patient’s condition gradually improved, and her oxygen requirement dramatically decreased without starting glucocorticoids. The patient was discharged on room air with mild residual symptoms and scheduled for follow-up after four weeks. The patient was completely asymptomatic on the follow-up visit, and her repeated CT thorax showed almost complete resolution of the previous interstitial lung changes (Figures 4-5).

## Discussion

Although rare, nitrofurantoin-induced lung injury is one of the most commonly reported pulmonary drug toxicities, reflecting the increased popularity of the drug [5]. The vast majority of patients (85-95%) who present with pulmonary reactions to nitrofurantoin are women, likely due to their higher prevalence of UTIs and long-term nitrofurantoin exposure [6]. Both acute and chronic reactions have been reported, with the chronic form being less frequent and more in the elderly population [7]. Acute and chronic types of nitrofurantoin-induced lung injury result from different pathogenic mechanisms; hypersensitivity reaction is the dominant feature in acute disease, while chronic reactions may result from either a cell-mediated or toxic response [6]. Different histopathologic patterns have been described, typically interstitial inflammation (often eosinophilic) in acute reactions or nonspecific interstitial pneumonia in chronic reactions [8]. Other patterns have also been described, including organizing pneumonia, chronic eosinophilic pneumonia, and desquamative interstitial pneumonia [9-11].

The clinical presentation of nitrofurantoin-induced lung injury is variable, warranting an extensive evaluation, and it often makes the diagnosis more challenging. Acute reactions usually present with fever, dyspnea, and cough that begin within hours to a few weeks after the first dose. In chronic reactions, the most common symptoms are an insidious onset of dyspnea, cough, and fatigue, which appear after at least one month or even years after initiation of treatment [5]. Physical exam in both forms is nonspecific and may show fine inspiratory crackles, respiratory distress, or nothing [12].

The evaluation begins with the usual work-up for new interstitial lung disease with a focus on detailed history to reveal the culprit drug or other exposures, exclude other conditions with a similar presentation, and assess the severity of respiratory impairment. Laboratory tests, radiological diagnostics, and BAL are typically nonspecific yet are essential to exclude other possible causes. Peripheral blood eosinophilia and high erythrocyte sedimentation rate (ESR) are commonly reported, as well as positive autoimmune serology, which may not indicate a rheumatic disease [6,13]. The most frequently reported findings in CXR (94%) are bilateral and basal lung infiltrates [13]. High-resolution CT may demonstrate bilateral ground-glass attenuation, irregular linear opacities, bibasal air-space consolidation, and fibrosis [4]. Lung function test generally shows a restrictive pattern with reduced diffusion capacity, and the BAL cellular count can reveal lymphocytosis, neutrophilia, or eosinophilia [6]. A lung biopsy is not usually necessary in a highly suspicious context of nitrofurantoin-induced lung injury [13].

The diagnosis is often made based on the compatible history of exposure to nitrofurantoin, radiological findings of interstitial lung disease, restrictive lung physiology, and exclusion of other causes, as in our case. Early recognition and disconsolation of nitrofurantoin is the cornerstone treatment that may be sufficient for clinical and radiological improvement [5,6]. The role of glucocorticoids has not been well established. In previous reports, glucocorticoid initiation in patients with severe respiratory impairment has resulted in rapid improvement [6]; however, this benefit may be inconsistent as resolution without glucocorticoids is common even in patients with severe pulmonary toxicity, similar to our case.

## Conclusions

Although nitrofurantoin-induced lung injury is well documented in the literature, it’s often misdiagnosed, resulting in delayed diagnosis, unnecessary treatment, longer hospital stays, and poor outcomes. This case, in conjunction with those previously reported, suggests there is a need for a high index of suspicion in patients being treated with nitrofurantoin. Thus, regular monitoring with early drug withdrawal at the first sign of pulmonary toxicity is crucial to avoid such serious and even fatal pulmonary reactions.

## References

[1] Gupta K, Hooton TM, Naber KG (2011). International clinical practice guidelines for the treatment of acute uncomplicated cystitis and pyelonephritis in women: a 2010 update by the Infectious Diseases Society of America and the European Society for Microbiology and Infectious Diseases. Clin Infect Dis.

[2] Fisher H, Oluboyede Y, Chadwick T (2018). Continuous low-dose antibiotic prophylaxis for adults with repeated urinary tract infections (AnTIC): a randomised, open-label trial. Lancet Infect Dis.

[3] Holmberg L, Boman G (1981). Pulmonary reactions to nitrofurantoin. 447 cases reported to the Swedish Adverse Drug Reaction Committee 1966-1976. Eur J Respir Dis.

[4] Weir M, Daly GJ (2013). Lung toxicity and nitrofurantoin: the tip of the iceberg?. QJM.

[5] Kabbara WK, Kordahi MC (2015). Nitrofurantoin-induced pulmonary toxicity: a case report and review of the literature. J Infect Public Health.

[6] de Zeeuw J, Gillissen AG (2018). Nitrofurantoin-induced pulmonary injury. UpToDate.

[7] Tatley M (2002). Pulmonary reactions with nitrofurantoin. Prescriber Update.

[8] Mendez JL, Nadrous HF, Hartman TE, Ryu JH (2005). Chronic nitrofurantoin-induced lung disease. Mayo Clin Proc.

[9] Cameron RJ, Kolbe J, Wilsher ML, Lambie N (2000). Bronchiolitis obliterans organising pneumonia associated with the use of nitrofurantoin. Thorax.

[10] Martins RR, Marchiori E, Viana SL, Grillo Júnior LS, Capelozzi VL, Valença LM (2008). Chronic eosinophilic pneumonia secondary to long-term use of nitrofurantoin: high-resolution computed tomography findings. J Bras Pneumol.

[11] Bone RC, Wolfe J, Sobonya RE, Kerby GR, Stechschulte D, Ruth WE, Welch M (1976). Desquamative interstitial pneumonia following long-term nitrofurantoin therapy. Am J Med.

[12] Karmali R, Stawitzky K, Srisethnil I, Simpson K (2020). A rare case of nitrofurantoin-induced acute lung injury. Clin Case Rep.

[13] Almeida P, Seixas E, Pinheiro B, Ferreira P, Araújo A (2019). Consider nitrofurantoin as a cause of lung injury. Eur J Case Rep Intern Med.

